# Do We Practice What We Preach? A Mixed Methods Study of Stress in Stress Experts: Implications for Transfer of Awareness and Learning

**DOI:** 10.1002/smi.70064

**Published:** 2025-06-20

**Authors:** Sarah E. Crozier, Anna Sutton, Sarah‐Jane Lennie, Cary L. Cooper

**Affiliations:** ^1^ Department of People and Performance Faculty of Business and Law Manchester Metropolitan University Manchester UK; ^2^ Psychology Department University of Waikato Hamilton New Zealand; ^3^ Anglia Ruskin University (ARU) Chelmsford UK; ^4^ Alliance Manchester Business School The University of Manchester Manchester UK

**Keywords:** awareness, education, expert status, identity, qualitative, stress

## Abstract

This two‐phased, mixed methods study develops our understanding of how knowledge, education and awareness about workplace health phenomena is utilized by experts and applied to their own working lives, through a study that explores how stress is transferred and applied in the management of one's own experience of stress. Phase one gained quantitative data from a sample of 118 stress experts across 18 countries, and phase two used qualitative data from life histories interviews and focus groups with 14 stress experts who had also participated in Phase 1. Phase one found that stress experts experience less occupational stress than a norm group. The number of years experts have been researching stress does not influence the stress‐wellbeing relationship. Instead, the greater the belief in their expertise influence, the better their wellbeing, and this effect is independent of the stressors they experience. Phase two built sequentially on this to explore experts' reflections regarding the management of their own stress and the influence of their expert knowledge. Narrative thematic analysis was undertaken to provide discursive insights that captured appraisal of learning and framing of stress experiences. We provide conceptual and practical contributions to further our understanding about how expert status in health impacts outcomes and how this wider learning has theoretical and practical impacts. We show how stress beliefs impact upon behaviors, emotions and cognition. We conclude that awareness and knowledge in itself is not always powerful enough to shape outcomes, and our data evidences how spirals of learning interact with environment and context over time through rich narratives that chart reflection on the development and maintenance of expert status.

## 1 Introduction

Do as I say, not as I do?

In many disciplines, awareness and education about psychologically healthy workplaces is a precursor for optimisation of positive outcomes. In this study, we seek to learn more about how this learning is applied, and do so through an exploration of the stress experiences of stress experts. Do stress experts practice what they preach? And why does it matter? Knowledge and awareness of stress at work is regularly advocated as a means of bringing about positive change to employee health and well‐being, yet there is a distinct lack of evaluation of this proposal (Nielsen and Shepherd 2022). This is especially interesting given how little we know about the ‘practice what one preaches’ ethos which is expected of busy and overworked psychology practitioners in their capacity as gatekeepers for promotion of a positive workplace culture (While 2015), or for managers and leaders more broadly in leading by example (Kranabetter and Niessen 2017).

There is a long history in the wider psychology literature and in the stress and health field that promotes heightened awareness and education as important in the optimisation of stress and well‐being outcomes (Nielsen and Randall 2013) and a growing recent interest in exploring phenomena such as mindfulness and awareness states (see e.g.: Choi et al. 2022; Jamieson et al. 2022). Paradigms in stress management assert the need for information provision concerning the factors that can cause stress at work, and promote the visibility of stress phenomena in working to mitigate against negative consequences at an individual and organisational level. Paradoxically, research across different occupations tells us that health professionals may not be particularly protective of their own physical and psychological health despite their heightened knowledge and experience (Harrison 2008). In this paper, we aim to explore the impact of stress experts' awareness and education on their stress levels and examine how stress experts may mitigate the effect of stressors on outcomes in their own lives. We do this by appraising the role of expert status in operating between the exposure to stressors and stress outcomes. In so doing we add to the theoretical literature on the mechanisms that underpin how awareness and knowledge are transferred in making sense of one's stressful encounters, and provide theoretical and practical contributions for the role of experts in maintaining positive organisational health climates.

### 1.1 Stress Management: Awareness Is a Good Thing?

Interventions in stress and health center on a need for increasing the interplay between individual awareness and organisational commitment in the implementation of strategies for workplace health protection, and this approach has informed policy and legislation for organisational health initiatives globally (HSE 2022; Richardson and Rothstein 2008). Despite this widely accepted view, there is more to learn about the individual factors that shape the success of stress management initiatives (Nielsen and Randall 2013) where further evaluation is required to fill gaps in our understanding of how learning about stress is transferred and applied when one encounters it in real life (Nielsen and Shepherd 2022; Nielsen et al. 2022).

The means by which education and awareness are acquired, enacted in practice, and reflected upon by the recipients of stress knowledge are not yet fully understood. Although there is a longstanding theoretical understanding of the appraisal of stressful events (Dewe et al. 2010), and the individual differences that can manifest as stress moderators in appraisal across broad psychological characteristics (see, e.g., Tsai et al. 2019) there is less work on the mechanisms at play in the application of specific education and awareness mechanisms (Sutton and Medvedev 2023).

There is however an emerging stream of work that examines how the individual reflects directly upon their own stress experience. Richardson and Rothstein (2008), in their meta‐analysis of the success of stress management interventions found that the most impactful techniques for minimizing problematic stress outcomes were those that utilized cognitive‐behavioral programmes as an ‘active’ strategy where individuals' cognition and appraisal are central to managing challenge. This is echoed in other recent work, for example, Tong et al. (2021) found that high mindful awareness as a cognitive skill set was associated with better coping and well‐being outcomes. Recent work has synonymized mindfulness with primary appraisal (Jamieson et al. 2022). Similarly, other work is concerned with stress mindset (see e.g. Ben‐Avi et al. 2018). Casper et al. (2017) found that those with a ‘positive stress mindset’ were able to employ constructive coping in anticipation of workload increases, and recent work has developed methods for the measurement of awareness itself (Sutton and Medvedev 2023). Equally important is how one's stress beliefs about self may spillover into perceptions and expectations regarding the stress that others are under (Ben‐Avi et al. 2018).

Other scholars have worked to unpack the complexities in the success of stress management initiatives as a function of the beliefs of those encountering stress. Jennings‐ Black and Britt (2022) explore pertinent constructs such as impression management as influencing the outcomes of stress management interventions. How one views stressful encounters may impact their engagement with and enactment of the skills and knowledge contained within an intervention program. For example, if the presence of stress in one's work life is deemed helpful to perpetuation of their image as a successful or busy individual, they may be less inclined to engage with strategies for its reduction. Other work is concerned with understanding the cognitive mechanisms that sit below workaholism or overwork behavior (Kirrane et al. 2018). Thus, there is a building interest in extending the conceptual basis for reflection upon interpretation of one's response to stressful events beyond the appraisal of the stressor itself (Sutton and Medvedev 2023; Jamieson et al. 2022).

In sum, although there is an appetite for exploration of different cognitive mechanisms that shape stress appraisals at the individual level, it appears that no studies capture the ways in which people draw upon their expert knowledge as they acquire and use it alongside stressful life events. Yet, there is an identified need for further understanding of how health and well‐being initiatives lead to changes in terms of behaviors, cognition and emotion, and how context and environments are conducive to the utilisation of psychological knowledge (Nielsen and Shepherd 2022). This is echoed by other researchers, where; “no studies to our knowledge have sought to more broadly assess personal beliefs about the experience and perceived social value of a high level of stress, specifically operationalized as a high workload” (Jennings‐ Black and Britt 2022, 2) yet we know mental preoccupation with work can have impacts on health and support needs (Eib et al. 2018).

### 1.2 Expert Status: Practicing What One Preaches?

Expert status is held by those who have “qualifications, track record and experience” (Burgman et al. 2011, 1) and social expectations of expert status rely on using such skills to produce effective outcomes for others (Walton et al. 1997, in Burgman et al. 2011). There is a body of work that suggests people are drawn toward careers that mirror issues they have prior experience of or a disposition toward; the ‘self‐selection’ of roles (Cable and Judge 1996), and this is seen to bring about damaging health behaviors in certain professions. For example, some research suggests that medical doctors are not very good at protecting their own health (Harrison 2008; Feeney et al. 2016). On a related theme, recent work has examined constructs concerned with work as a ‘calling’ (Zhou et al. 2023), which can act as an important identity component and can impact the ways in which an individual fosters a psychological attachment with their role. For example, Andel et al. (2022) in their study of paramedics found that work that is deeply woven with a sense of purpose and a calling may result in job‐related challenging emotive circumstances being more keenly felt by participants, and lead to heightened detrimental impacts such as mental exhaustion as a function of negative rumination.

A small number of research publications have addressed the prevalence of occupational health related difficulties in practitioners who have expertise in a range of psychological health areas. Galeazzi et al. (2003) for instance, explore the motivations for career choices in psychiatry and find that individual experience of and exposure to mental ill health is a determining factor. There is increasing interest in the impact for professionals of working with individuals with psychological health difficulties. Indeed, studies have suggested secondary trauma and increased stress arise as a function of exposure to others' challenges and experiences (Livanou et al. 2024; Gartner 2016; Preston 2022). This also helps to advocate for an exploration of how experts' experiences of stress are potentially both independent from and dependent upon the interactions with those they work with, and the subject matter they are exposed to, and therefore may be abundant both for themselves and within their work environment. At the same time, there is a potentially conflicting need to preserve one's own health as a health expert in order to lead by example, perpetuate a positive organisational culture and climate, and manage a credible professional identity (While 2015). Further, though not examining expert status per se, Kranabetter and Niessen (2017) discuss the self‐regulatory health awareness of leaders and its impact on subordinates. They find interesting potential for social learning and role modeling from managers in bringing about positive health behaviors in others as a function of the leaders' own attitudes, knowledge and reflections regarding health. Moreover, expert knowledge should potentially act as an important learning experience to preserve positive health outcomes. Sawhney et al. (2018) for example, draw on work recovery systems as an important focus and suggest that mastery experience is an influential means to stress recovery. Mastery experience refers to the acquisition of learning and knowledge. In our study, we frame expert knowledge as a potential mastery experience. Thus, there is a paradox in the literature base on expert status. On the one hand, we would expect it to help shape positive outcomes‐especially given the purpose of education and awareness as critical for stress management, and on the other, as we have seen here, it can be potentially damaging.

### 1.3 Pluralistic Enquiry in Stress and Health Research

We argue that in order to learn more about the stress experiences of stress experts and the factors that impact their lived experience of stress, both quantitative and qualitative enquiry are important, and we employ both in this study. For some time, researchers have addressed the need for multiple perspectives in building our learning of underexplored phenomena within stress research. This includes analysis of multiple data sources from traditionally opposed singular research methodologies (Lazarus 2000; Mazzola et al. 2011; Crozier and Cassell 2016) in understanding both the intra‐ and inter individual differences that are important in unearthing complexities in the process of stress experiences. In recent years, mixed‐methods approaches to the study of work and stress have gained significant traction especially as a means to strengthen the existing contribution from more traditional quantitative approaches. For instance, mixed methods have contributed to capturing the nuances in the evaluation of outcomes (Nielsen et al. 2022) especially in terms of exploring the processes that sit below the success of stress initiatives. There is also recognition that qualitative methods allow the ‘why’ alongside the process between interventions and outcomes to be explored (Nielsen et al. 2022) and allow for a consideration of the contextual factors that may be missed in more reductionist designs. Wilhelmy and Köhler (2022) cite qualitative methods as valuable for capturing the “underlying mechanisms and processes creating individuals' behaviors” (p162) and this is a view shared in process‐orientated stress research (Crozier and Cassell 2016).

### 1.4 Theoretical Framework

We bring together a number of important theoretical strands and integrate them into a framework. We note the requirement for a multi‐level understanding of how learning is transferred from the environment from which it is acquired to real life, and how it is drawn upon and revisited over time in response to different stressful events and environmental stimuli (Nielsen and Shepherd 2022).

In addressing this research gap, we use expert status as a proxy for the transfer of learning that is suggested as an important gap in our knowledge of stress interventions (Nielsen and Shepherd 2022). We suggest that expert status is a reasonable proxy for examining the transfer of learning as stress experts hold an exemplary amount of knowledge and awareness. We draw on elements within the broad premise of the recent ITTEM (Integrated training transfer and evaluation model) (ibid) where; “learning may be an important precursor to changes in mental health and wellbeing outcomes” (p378). We seek to unpack the elements of learning for the stress expert and do this through a consideration of stages 4 and five of the ITTEM. The earlier phases of the ITTEM (1–3) are concerned with the training itself and the immediate transfer into the work environment, but as experts have acquired their knowledge earlier and over a significant period of time, we explore how the later stages (4–5) of the model are enacted. These stages focus on how some time after training (in our case acquisition of expert status), the participant utilizes their learning to bring about and maintain “sustained changes in emotions, cognitions and behaviors” at stage 4 (386) which then—if successful‐lead to positive impacts in stress and health outcomes at stage 5. It is acknowledged that these stages of the model are likely to be cyclical and repeated numerous times.

Importantly, we echo a need for further work to understand what happens between each level of the model and how learning is revisited in light of the extent to which earlier learning successfully impacts outcomes for the individual. This is referred to as a spiral effect and is comprised of ‘complicated patterns’ (Nielsen and Shepherd 2022, 382). In their model, a number of factors are likely to influence the extent to which positive outcomes are attained. These include how relevant the learning is for one's work environment; whether the individual intends to use the knowledge; the amount of support they have; and opportunities to practice etc. In building on this we seek to explore the beliefs about expert status as an influencing factor.

**FIGURE 1:**
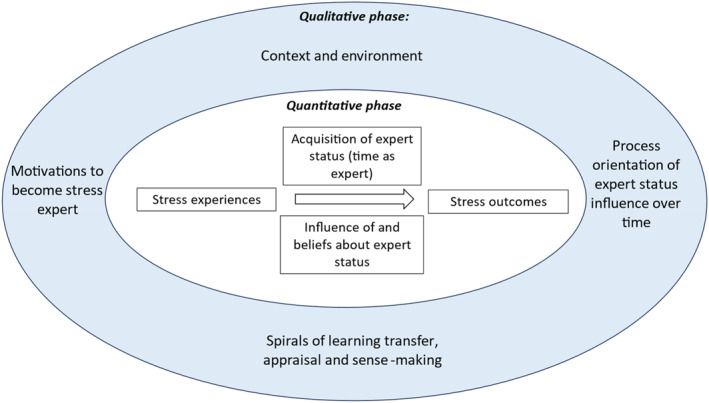
Theoretical framework: The influence of expert status on stress experiences.

In linking the theoretical notions together to form our conceptual framework, we provide a visual representation that positions a model to depict the influence of expert status on experts' stress experience and outcomes (Figure 1).

First, the inner core of the model represents the quantitative phase of our study, where we propose hypotheses to test the predicted relationship between expert status and stress experiences. We suggest expert status will influence experts' reflections on their own stress awareness and experiences as suggested in stress frameworks that link personal characteristics to stress outcomes (e.g. Dewe et al. 2010). These outcomes are many and varied, from physical symptoms such as increased heart rate or cortisol, through psychological effects like depression and anxiety, to specifically occupationally‐relevant outcomes such as organisational commitment, burnout or job satisfaction (e.g. Richardson and Rothstein 2008). However, primary outcomes can be measured succinctly using the ASSET questionnaire (Faragher et al. 2004), which evaluates the employee's organisational commitment, physical health and mental health, and we focus on these outcomes in this study where these variables are measured in our quantitative phase of data collection. Our hypotheses are.

Hypothesis 1 Stress experts will experience less occupational stress than a non‐expert norm group.

Hypothesis 2 Stress experts will experience fewer detrimental stress outcomes than a non‐expert norm group.

Hypothesis 3 Expert status will have a direct effect on stress outcomes.

Hypothesis 4 Expert status will moderate the relationship between stressors and stress outcomes.

Sitting outside of this inner core of our framework are a variety of further important influences that we seek to explore through the qualitative phase in study 2. We suggest that motivations to develop expert status and begin working within the field are important as per the sparse literature base that links some form of predisposition or topical interest with a self selection of careers (e.g. Galeazzi et al. 2003). This motivation therefore shapes acquisition of expert status and beliefs about it. The importance of context and environment as derived from the IITEM, plus how the expert interacts with their environment through ongoing sensemaking and appraisal works to shape continual learning that in turn reinforces or reshapes motivations, awareness and experience over time. Taken together this helps to position stress expert status as a process‐ oriented experience where beliefs and experiences interact with expert status to shape outcomes.

In sum, our model positions stress expertise as a process‐orientated mastery and learning experience (Sawhney et al. 2018) that is situated as both a personal resource for the stress expert, and as part of a professional identity that evolves over time as it interacts with contextual and environmental factors. Its deployment is influenced by the cognitive beliefs about the influence of such knowledge and the purpose it can serve in helping both the experts' own stress and that of those that they support within their work (Andel et al. 2022). We suggest that pluralism in methodology is required to both capture the quantitative measurement of the prevalence of stress within the expert population alongside narrative enquiry to elucidate the process concerned with its enactment and the ways in which it is reflected upon and made sense of to allow the capture of complexities in the spiral of learning transfer over time. Watson et al. (2018)'s work on well‐being and learning posits individual factors working alongside organisational factors in learning and its connection to well‐being outcomes. Therefore, our model includes reference to individual acquisition of knowledge but also how this is drawn upon in response to the context and environment, and as a function of motivations for working in the stress field. Stress beliefs may flux and change as a response to environmental expectations about stress identity for the expert (Andel et al. 2022; While 2015; Jennings‐ Black and Britt 2022; Livanou et al. 2024).

In summary, we present a two‐phase mixed methodological account of stress experiences within the stress expert population. The aims are two‐fold. First, we explored whether stress experts experience higher or lower stress experiences than non‐expert populations, and second, we aimed to further add to the pluralistic methodological exploration of sense‐making in stress and coping research. For both phases, the goal was to appraise the influence of expert status in operating between exposure to stressors and their consequences.

## 2 PHASE 1: Using a Quantitative Approach to Understand Stress Experts' Stress

### 2.1 Methods and Measures

Ethical approval was obtained through Manchester Metropolitan University's ethics committee for both phases and participants gave informed consent to participate. Phase one involved the design of a survey utilizing the ASSET (A Shortened Stress Evaluation Tool; Faragher et al. 2004) and some exploratory items pertaining to expert status and its influence on stress experience (e.g. “I am more likely to experience stress at work due to my knowledge of occupational stress; Having expert knowledge about occupational stress enables me to cope better with my own stress”).

Respondents were invited to complete an online questionnaire consisting of demographic questions including nationality, age, sex, marital status, dependents, working status of partner, as well as the number of years the respondent had been researching stress. Next, the ASSET questionnaire which has three sections. The first section measures attitudes toward the organisation on a six‐point scale and the ASSET manual reports two subscales: organisational commitment toward the employee and employee's commitment to the organisation. Second, there are 17 questions about the respondent's physical and mental health, measured on a 4‐point frequency scale (from *never* to *often*). Two subscales Physical Health and Psychological Wellbeing are created from these items, with higher scores indicating increased problems in these areas. The third section (perceptions of your job) asks respondents to report on potential stressors in their job (e.g. *I am troubled that I work longer hours than I choose or want to*.) A six‐point Likert agreement scale is used, such that higher scores indicate greater stress. The ASSET manual reports six subscales in this section: Work relationships, Work‐life balance, Overload, Job security, Control, Resources and communication, and Aspects of the Job. In addition, Pay and Benefits is measured by a single item.

Finally two different measures of ‘stress expertise’ were used in this study. First, the number of years the respondent had been researching stress was assessed on a five point scale: < 2 years, 2–5 years, 5–7 years, 7–10 years and > 10 years. Second, eight items were developed by the authors which asked respondents to indicate the extent to which they believed that their research into or knowledge about stress might influence their experience of stress or use of coping strategies. (e.g. *Having expert knowledge about occupational stress enables me to cope better with my own stress*.) These items were scored on a six‐point agreement scale such that higher scores indicated greater influence. Cronbach alpha for this scale was 0.74.

### 2.2 Sample

A database of stress experts was constructed based on a literature search of published academic journals in occupational health psychology, organizational psychology, and stress and health. Authors of papers on stress at work were entered into the database giving a total of 527 individuals. Criteria for inclusion were the publication of an academic journal article in the stress field within the last 5 years. Participants were emailed an invitation to participate which included a link to the online survey.

The final sample achieved a 22.4% response rate (*n* = 118), was 59% female and 94% of respondents worked full‐time. The majority (47%) were American, 17% British, 20% other European nationalities and the remaining 16% other nationalities. Forty percent of respondents had been researching stress for 10 years or more and the mode age group was 41–50 years old. Eighty percent of the sample were married or living with a partner, and two thirds of these partners were in work (77% of them full‐time).

### 2.3 Results

ASSET sub‐scales were calculated using the ASSET manual guidelines, and means, SDs and alpha reliabilities are shown below in Table 1. Cronbach alphas were acceptable for all scales except WLB and control, where they were just below 0.7. Given their status as established scales, however, these two were retained for the analysis. The ASSET scale of ‘Aspects of the job’ had reliability of only 0.48 and was therefore dropped from further analysis.

**TABLE 1:** Descriptive statistics and correlations for the scales used in this study.

	Mean	SD	Α	1.	2.	3.	4.	5.	6.	7.	8.	9.	10.	11.	12.
1. Time researching stress	3.69^a^	1.29	^b^												
2. Expertise influences stress	3.25	0.74	0.74	0.04											
3. Work relationships	16.93	6.77	0.82	−0.08	0.06										
4. Work life balance	12.02	4.32	0.68	−0.07	−0.05	0.34^**^									
5. Overload	12.23	4.61	0.76	−0.13	−0.03	0.30^**^	0.55^**^								
6. Job security	10.18	4.20	0.70	−0.23^*^	0.08	0.34^**^	0.26^**^	0.26^*^							
7. Control	9.24	3.77	0.68	−0.08	0.09	0.66^**^	0.22^*^	0.24^*^	0.41^**^						
8. Pay	3.08	1.67	^b^	−0.07	−0.01	0.22^*^	0.01	0.19	0.20^*^	0.30^**^					
9. Resources/communication	9.32	3.70	0.63	−0.01	−0.05	0.65^**^	0.22^*^	0.39^**^	0.29^**^	0.65^**^	0.32^**^				
10. Org commit to Emp	21.39	5.32	0.85	0.07	0.01	−0.32^**^	−0.08	−0.13	−0.01	−0.42^**^	−0.21^*^	−0.44^**^			
11. Emp commit to Org	17.73	4.26	0.85	−0.01	−0.03	−0.16	0.06	−0.03	0.10	−0.32^**^	−0.16	−0.30^**^	0.82^**^		
12. Physical health	12.23	4.11	0.78	−0.27^**^	−0.29^**^	0.29^**^	0.22^*^	0.34^**^	0.18	0.27^**^	0.31^**^	0.32^**^	−0.23^*^	−0.09	
13. Psychological wellbeing	21.31	7.73	0.93	−0.23^*^	−0.28^**^	0.39^**^	0.37^**^	0.41^**^	0.25^*^	0.43^**^	0.29^**^	0.46^**^	−0.30^**^	−0.18	0.75^**^

^a^ Note that this is the mean on a five‐point scale, not the mean number of years.

^b^ Single item measure.

^*^ *p* < 0.05.

^**^ *p* < 0.01.

The seven scales measuring perceptions of stressors were positively correlated with each other and with health and wellbeing, indicating that greater stress is associated with increased health and wellbeing problems. The two measures of attitudes, organisational commitment to the employee and employee commitment to the organisation, were very highly correlated and showed some negative correlations with stressors, indicating that commitment tended to be higher when stressors were low.

In order to evaluate the stress levels and outcomes of stress experts, their scores were compared with the ASSET *professional and managerial* norm group, using single sample *t*‐tests against the norm mean for each scale (as in Tytherleigh et al. 2007).

In general, stress experts are less stressed than the managerial/professional norm group. Stress experts report better work relationships, more job security, more control and better resources/communication. There were no differences between the groups for work‐life balance, overload and pay. Hypothesis one is partially supported.

In terms of attitudes at work, stress experts indicated that their organisations demonstrated greater commitment to them than the norm group did, but there was no difference in the employees' commitment to the organisation. Stress experts reported better physical and psychological health than the norm group. Hypothesis two is partially supported.

Correlations between the two commitment subscales as well as between the two health outcome subscales were very high. Although they did not reach the *r* = 0.9 cut‐off suggested by Tabachnick and Fidell (2018) as a cause for concern with multicollinearity, we conducted factor analysis to determine whether the subscales could be statistically distinguished in this sample.

Principal Components Analysis was run on all of the commitment items. The scree plot indicated that a single factor provided the best solution, with an eigenvalue of 5.5 and explaining 61% of the variance. All items were significantly positively correlated (majority *p* < 0.001) and had their highest loadings on the first component extracted. A second component accounted for an additional 12% but did not distinguish between the two proposed ASSET subscales (organisational commitment to the employee and employee commitment to the organisation). In this study, therefore, we combined these items into one scale, called *commitment*. This new scale had a Cronbach alpha of 0.92 Table 2.

**TABLE 2:** *t*‐test results to compare expert stressors and outcomes with norm group.

	Norm group		Experts		*t*‐value
	Mean	SD	Mean	SD	
Stressors					
Work relationships	20.26	7.11	16.93	6.77	−4.92***
Work‐life balance	12.87	4.42	12.02	4.32	−1.97
Overload	12.59	4.40	12.23	4.61	−0.78
Job security	11.04	3.63	10.18	4.20	−0.21*
Control	13.30	4.72	9.24	3.77	−10.77***
Resources and communication	13.12	4.39	9.32	3.70	−10.28***
Pay and benefits	3.31	1.74	3.08	1.67	−1.38
Attitudes					
Org commit to employee	19.12	5.28	21.39	5.32	4.25***
Emp commit to Org	17.08	3.87	17.73	4.26	1.51
Outcomes					
Physical health	13.32	1.04	12.23	4.11	−2.62**
Psychological wellbeing	23.07	7.04	21.31	7.73	−2.26*

The same procedure was followed for the two wellbeing subscales (physical health and psychological wellbeing). Again, PCA indicated that a one factor solution was the best fit for the data, with an eigenvalue of 8.3, explaining 49% of the variance. All items were significantly positively correlated (the majority with a significance of *p* < 0.001) and all items except one (*panic attacks*) had their highest loading on this first component. We therefore combined all these items into an overall *wellbeing* scale. This new scale had a Cronbach alpha of 0.93.

PCA of the stressors items (not including ‘job overall’ items which had already been excluded in previous analyses) also indicated a single factor solution from the scree plot, with an eigenvalue of 8.85 and explaining 24% of the variance. All items were positively correlated, the majority with a significance < 0.05. A scale created from all items (called *stressors*) had a Cronbach alpha of 0.89.

### 2.4 Moderation Analysis

To assess the influence of expert status and explore the extent to which the relationship between stressors and wellbeing is impacted by expert status, hierarchical multiple regression analyses were conducted. Controls were entered in the first step (sex, age, marital status, number of dependent children, work status of partner) and accounted for a significant amount of variance in wellbeing, *R* ^2^ = 0.38, *F*(5.72) = 6.99, *p* < 0.001.

In the second step, the two predictors were entered: stressors and the expert status measure. The analysis was run twice: once with ‘number of years researching stress’ as the measure of expert status and once with the ‘expertise influences stress’ scale.

Adding the *stressors* and *time researching stress* variables resulted in a significant increase in the amount of variance in wellbeing ΔR^2^ = 0.14, *F*(7.70) = 8.88, *p* < 0.001. The standardised *β* for time researching stress was not significant, however.

Adding the *stressors* and *expert influence* variables resulted in a significant increase in the amount of variance in wellbeing Δ*R* ^2^ = 0.20, *F*(7,70) = 10.90, *p* < 0.001. The standardised *β* for expert influence was −0.238 (*p* < 0.01), indicating that expertise is associated with increased wellbeing.

In the final step, the interaction term between stressors and expert status was added to the model. In neither case did the interaction term significantly improve the model fit. This indicates that expert influence is directly related to wellbeing, rather than acting as a moderator of the stress–wellbeing relationship. Hypothesis three is supported. Hypothesis four is not supported.

### 2.5 Phase 1 Summary

Analyses indicate that stress experts experience less occupational stress and have better wellbeing than the norm group. Analyses indicate that the number of years stress experts have been researching stress for does not influence the stress‐wellbeing relationship. Instead, it appears to be the experts' belief in the extent to which their expertise and knowledge will influence their stress levels that is significant. The greater the belief in this expertise influence, the better their wellbeing, and this effect is independent of the stressors they experience at work. This contributes to our understanding of how learning and changes in cognition are important in shaping outcomes when we evaluate the influence of training or awareness (Nielsen and Shepherd 2022).

## 3 PHASE 2: Qualitative Interviews and Focus Groups With Stress Experts

### 3.1 Method and Measures

A semi‐structured interview schedule was designed with the aim of allowing a degree of freedom for participants to share their experiences of stress as a function of their expert status. Questions were constructed that sought a life histories narrative in order to map previous and current experience and ascertain beliefs about the connections between them (Mazzetti and Blenkinsopp 2012). The interviews began by asking participants to recount their experience of beginning to work in the stress field in terms of their motivations and circumstances. Questions then asked participants to reflect upon their perceptions of their own stress and coping. A question was then posed regarding the influence of expert status and experts were asked to share their own beliefs regarding the impact of their expert status. Questions across these three segments are mapped to an examination of the three levels in understanding the influence of intervention (in this case stress experts' expertise)—learning, changes in cognition and emotion, and outcomes in terms of own stressful experiences (Nielsen and Shepherd 2022).

### 3.2 Sample

All database participants were sent an email from the research team informing them of phase two and asking for their volunteered participation. Fourteen participants responded and took part in either online focus groups or one to one interviews (some of which were virtual and some face to face). Demographic information for participants of this part of the study is not reported in order to ensure anonymity, but participants were from six different countries, both male and female, and had been researchers or practitioners in occupational stress for both longer and shorter time frames, and therefore representative of the sample for Phase 1.

### 3.3 Analysis

A narrative thematic analysis was undertaken in order to allow both an examination at the idiographic (within person) and nomothetic (between person) levels as is typical in research that seeks to understand the process of a psychological phenomena (Crozier and Woolnough 2020). This allows a move away from one dimensional data into the temporal trajectory space given the emphasis on life/work histories (Spencer et al. 2021) where connections between experiences, their historicity and their impacts can be examined. This is especially meaningful when attempting to trace the process orientation of a phenomena.

We suggest that a telling of one's stress journey, here the sense‐making that stress experts apply to their own stress experience is suited to a narrative analysis. Narrative analysis has been widely used as a qualitative approach to capturing appraisal and subjectivity in action (Graci and Fivush 2016). It provides an opportunity to capture the stories surrounding an event or experience as they are represented by the subject and therefore allows lived experience of an issue to be preserved in the data presentation. This is important for uncovering stress experts' experience of their own stress and in extrapolating the impact of expert status as it allows a flexible, thorough and discursive approach to exemplification of the cognitive mechanisms that underpin how one accounts for their own experiences of stress and how their experience interacts with their framing and meaning as a function of their expert status. Indeed, Mason et al. (2019) explore the buffer effects of the way in which non‐experts with caring responsibilities frame a demanding experience in terms of self‐awareness, growth and adaptation. They state; “Narrative researchers have shown that people who coherently integrate difficult experiences into their life story tend to have better mental health” (101).

A template analysis (King 2004) was constructed as a matrix that presented each broad segment of data from the question set against emergent themes from each of the participants' responses. The question set was segmented to represent three core strands of enquiry, that reflect the notion of process in the stress experience. The first segment was concerned with the motivations for working in the field of stress, the second captured participants' own lived experience and awareness of stress, and the final segment appraised perceptions about expert status.

Data was first analyzed ideographically where each participants' account was transposed to a matrix singularly. Following this, broad themes across all participants were collated to form the final template (see Supporting Information S1). A summary of this is provided in table three. For the purpose of this paper idiographic templates are not provided in order to protect anonymity of participants given their proximity to the readership of the publication outlet and discipline. Similarly, participant quotations are not attributed to participant number to further preserve anonymity.

### 3.4 Phase 2 Findings

#### 3.4.1 Segment 1: Motivations for Becoming Stress Experts

Participants cited a number of circumstances that led to their involvement in the stress field. This was for a small number of participants unintentional and serendipitous such as stress emerging as an area of focus from other occupational research.

##### 3.4.1.1 Narrative: Personal Circumstances as Informing Career Choice

The most pertinent theme was personal stress exposure as informing career choice. The stress expert with lived experience of stress and trauma cited their experience, though troubling, as a pertinent tool for helping to build further their expertise and compassion in dealing with their own stressful experiences, and also in developing empathy and compassion for those they work with, echoing the calling literature (Zhou et al. 2023; Andel et al. 2022); Table 3

I do suppose it has been a bit of cycle really, because if it wasn’t for the extreme stress and trauma then I wouldn’t have chosen the career… but my understanding of the brain has allowed me I suppose to forgive myself for not having been able to manage stress…

**TABLE 3:** Narrative thematic analysis matrix.

Analytical segments	Emergent high level nomothetic narrative themes
Motivations for becoming a stress expert	Personal exposure as informing career choice
	Helping others
Experiences and awareness of stress	Stress experts are not immune to stressful experiences and damaging consequences
	The stress expert as overworking (I do not practice what I preach?)
	Reflecting on coping complexities
Beliefs about expert status	Stress knowledge and education as helpful
	Heightened awareness and expertise don't necessarily improve outcomes
	Identity and the disclosure paradox for the stress expert
	Learning from others

Other participants described how parallels between their own experience and personality became apparent some time after commencing their career;

But when you unwrapped it you realised it was the stress, the expectation and pressure that would be put on them…the stress of moving their families…it was several layers of stress, and so we unearthed that, but when I got there I started to think ‘hmm some of the interviews that I am hearing are me’ so you know, so that’s when the personal came in to it.

##### 3.4.1.2 Narrative: Helping Others

A vocation of helping others was cited as a common reason for working within the stress field, and this was considered as woven within identity and purpose (Andel et al. 2022). One participant noted their strong beliefs in social justice and a desire to help others alongside reflections about personality and fit with the discipline. Other participants noted their need to help others but a desire to do this in the occupational, rather than clinical sphere.

I used to gravitate towards other people’s crisis and stuff because I wanted to know that I had a role and a function, and I knew that I could take it and I could absorb the stress, well I thought that I could. I thought that that was my role, I had inherited it, this profession…

#### 3.4.2 Segment 2: Awareness and Experiences of Own Stress

##### 3.4.2.1 Narrative: Stress Experts Are Not Immune to Stressful Experiences and Damaging Consequences

Almost all participants cited experience of stressful encounters within their own lives and acknowledged stress as omnipresent, where they and others were not immune to exposure or detrimental impacts. Participants shared detailed accounts of psychological ill‐health as a function of stressful experiences and drew upon their learnings from prior traumatic or stressful situations in making sense of their stress and applying coping strategies;

When I had those two acute episodes of stress, I was alarmed that I could actually get to that point and that I had such a lack of self‐care, of course, I reflect upon the fact that three years ago when [several stressful life events took place]. I don’t know if I could have done very much about it, you know, it just happens. It’s just a situational acute stressor, but I guess day to day I can manage my stress I think much better now than I could three years ago.

##### 3.4.2.2 Narrative: The Stress Expert as Overworking (I do Not Practice What I Preach?)

There were many stories shared of the stress expert working long hours and taking responsibility for their own stress levels, despite an awareness of its potentially detrimental consequences. A number of complexities were examined in the narratives. First, an acknowledgment that sometimes exposure to stressful situations and overwork (Eib et al. 2018; Kirrane et al. 2018) as a conscious and voluntary choice for the stress expert, and a belief and cognitive framing that if one enjoys ‘work’ then it is not really ‘work’ at all;

And it is my fault sometimes…I know how hard it is and I hate this trite stuff when you say to people ‘oh you know, just stop work at five o’clock and have a life and this that and the other’ because you know how hard it is when you are deeply involved in what you are doing and passionate about it and so I sometimes feel a bit of a fraud really. But it is a difference between the psychological flow and the being in a trapped situation, if you are in the zone loving work, why wouldn’t you carry it on?

Reflections of overwork were also framed as flexibility preferences for the stress expert where once more beliefs and cognitive appraisal of these working arrangements as convenient were used as a mechanism for offsetting its potential for harm. This was considered alongside an awareness that the same way of working can be damaging for others, illuminating complexities in how self‐regulatory health awareness is transferred or has the potential to influence others (Kranabetter and Niessen 2017);

…if I want to read my emails and send some emails on a Sunday at eight o’clock, so that I can get them out of my head, I will… you know again, with growing knowledge of impact on other people, role models whatever, you have got to think about the impact on the recipient as well…,so I am much more aware of that, and the signals that you are giving to other people.

Similarly, participants cited their belief about personality dimensions as impacting their choices about work and life integration (Kirrane et al. 2018). Stress experts emphasized taking responsibility for their overwork, and in a number of examples cited it as a voluntary activity. Sometimes overwork was positioned as a coping strategy as distraction from bereavement and trauma;

I do get stressed, I use work as a coping mechanism and if anything happens to me, you know like really bad things, like my mum died, and it was a very traumatic situation …if things become tough I throw myself into work and I become pretty much incommunicado and I like to insulate myself from the outside world, you know, which I know is not good… I work long hours, I am obsessed by work and I use it as coping, and I know that is not healthy at all.

##### 3.4.2.3 Narrative: Reflecting on Coping Complexities

Stress experts reflected in detail about the complexities in coping with stress, and often punctuated their experiences with reference to ongoing learning. It was clear that meaning about coping was ascribed as a function of topic‐related theoretical knowledge, and the context was deemed pertinent in the decision framework employed around coping. Likewise, participants cited a number of healthy or adaptive coping strategies from exercise and support‐seeking to mindfulness practices and healthy lifestyle choices. Similarly, beliefs about stress and its challenges were evident in cognitive framing of stressful encounters. Stress experts shared changes in coping as a function of learning and perseverance over time. This was appraised alongside stressors fluctuating as career circumstances changed. Interestingly, this participant for example noted that transfer of learning and success of that transfer was time‐consuming and required a lot of commitment;

Even just yesterday I found the stress coming out, just because the job that I am doing now, I am inheriting a lot more responsibility, and I have got more of a profile than I was ever prepared for. And I think that is starting to have a bit of an effect on me which is good, and I will adapt. But there is an element of discomfort in that, and I was very much feeling that…but I thought, ‘no what I will actually do…I will make sure that I get some time and some space tomorrow morning’, and I got up extra early today and I did that, I set myself to get my brain in the right gear…and it doesn’t take so much, whereas before it was so difficult, I found the mindfulness stuff so hard. I had almost given up on myself after about four or five years…but just persevering, and things have really, really changed.

Some perceptions centered on the transient nature of stressful experiences and explored how belief and emotional framing regarding one's ability to cope can fluctuate frequently. This was especially important as an element of cognitive self‐talk that could appease the intensity of stress through conversations with oneself that supported the temporary nature of one's appraisal, affect and cognition as a function of ever‐shifting contextual factors (Spencer et al. 2021). These reflections also shaped learnings about stress methodology;

We are aware of the transient nature of coping ‐ one morning you will wake up and you will think ‘my gosh, there are all of these things that I have to do’ and it is like ‘tick, tick, tick’, and everything gets filed away beautifully, you know you are really productive and you do really well… and of course on that day you would rate yourself as being really good at coping with stress, really productive. And then the next day you wake up feeling, you know, a bit low and feeling as if the whole weight of the world is on you… I should be able to cope with this, but really that is tricky isn’t it? As we know with stress research that a lot of the measures that we use tend not to capture that because they are done by asking people for a general picture, you know ‐ how do you behave? How do you feel normally?

The interplay between the stress experts' knowledge, environment and context were highlighted in the selection of coping strategies, echoing an iterative spiral of learning (Nielsen and Shepherd 2022). Stress experts also highlighted the way in which one constructs and presents their stress persona as mechanisms for coping. This supports work on stress as a badge of honor (Jennings‐ Black and Britt 2022) and illustrated impression management (claiming to be busy so one is not given more work) as a function of stress disclosure.

#### 3.4.3 Segment 3: Influence of Expert Status

##### 3.4.3.1 Narrative: Stress Knowledge and Education as Helpful

Stress experts discussed the influence of their expert status in shaping their own experiences of stress. They asserted that knowledge and awareness were useful in order to move to a position of taking action;

I suppose if you have the ability to recognise your understanding of stress, then I guess you have the ability to, to know to do something about it. So, it is logical that you then take that step to do something about it. If you were from a position of ignorance, and you didn’t know why stress occurred and where it comes from, and you were just feeling stressed then without that understanding then you don’t feel like that you have got the tools to do anything with it.

Reflections on the utilisation of expert status in optimizing one's own health was also discussed in terms of its impact on others (Kranabetter and Niessen 2017);

…the more that I use my understanding in my so called expertise about the brain and trauma to demonstrate that it can have a positive effect the better, so I suppose managing my stress in a way isn’t just for me ‐ it is a way for demonstrating that things can be better for other people too, so the more that I do it the more that I am likely to be able to help other people and also for the people that live and work around me, I want to be a person that that is nice to be around.

##### 3.4.3.2 Narrative: Heightened Awareness and Expertise Doesn't Necessarily Improve Outcomes

Stress experts discussed nuances in how awareness and education impacted stress outcomes in navigating their own stress experiences. Interestingly, perceptions about inability to change one's environment and a lack of control were seen as determinants of poor outcomes despite expert knowledge;

So having studied up on all these things and done research on them I guess I was able to think about it in a more coherent kind of way… I would have to say that I am not sure that any of this did me a whole lot of good though, in terms of when there were times when I felt very stressed out in the work that I was doing. And even though I was aware of it, there were some times when I just felt that I wasn’t able to change what was happening so that in itself was one of the stressors.

##### 3.4.3.3 Narrative: Identity and the Disclosure Paradox for the Stress Expert

Stress experts outlined a number of problems in managing their own stress alongside their expert status;

But having high expectations of yourself is problematic because you feel that you shouldn’t be feeling like this and you should be able to cope with it in some ways, you know.

There were paradoxes for the stress expert in disclosing their own stress experiences with those they work with or help, and with their peers. On the one hand, lived experience of stress was seen as an important rite of passage to compound one's expert status, yet on the other hand, experts shared concerns that revealing one's stressful experiences may impact negatively on perceptions of their stress expertise. Stress experts discussed the notion of responsibility as intertwined with their expert status where a possible stressor in itself was the outward ability to practice what one preaches. This was considered important for a number of reasons. Similarly, participants expressed conflict between disclosing stressful experiences as authentic, and as potentially eroding the credibility of their expert status;

…once I gave a workshop…anyway, this person introduced me and said—‘[name] is going to talk about stress, and having had the pleasure of her at lunch I realise that she is the least stressed person I have ever met’. And I remember thinking ‘is this an insult?!’ and actually it was a compliment saying, you know, she obviously practices what she preaches… but the flip side is that I was doing a session last week and I used some bits of personal disclosure and in that I thought ‘I hope they don’t think that I am actually a very vulnerable or weak person’ … so there is this balance all the time and it can be quite tricky.

##### 3.4.3.4 Narrative: Learning From Others

Stress experts made direct reference to their expert status as offering the opportunity to interact with and learn from those they work with (Livanou et al. 2024). This evidenced ongoing learning spirals, where despite expert status as already established, it was revisited and built upon through interactions with one's experience and environment. One such learning was concerned with stress as a function of emotional reactions rather than a reaction to an anticipated stressor itself;

I mean working with loads of people over the years, my belief is that none of us are actually worried about anything, or stressed about anything, we are stressed by our emotional reaction to it. I mean, if you thought, I could cope with a marriage break up it is self‐efficacy, then I will be able to [cope] … I think we are more worried about our emotional response to things rather than the actual thing itself? …I suppose I do a lot of reflecting on it and what I learnt from it, what can I gain from it, how has it added to my tool kit.

Participants also noted how they learnt about their own identity as an expert from their interactions with other experts, especially in terms of norms for behavior and best practice. This was discussed in terms of observing other experts and reflecting on their own skill set and identity. Further, experiential learning was deemed imperative, and here one expert noted it as more powerful in interacting with their own responses to stress than their academic training as an expert.

### 3.5 Study 2 Summary

Through three analysis segments our data unearth narrative themes that explore stress experts' reflections about their own stress experiences and beliefs about their expert status. This captured the process orientation of their expert status through first examining motivations for participating in stress as a career path, before providing reactions to their own stressful encounters. Finally, different discourses about the impact of expert status were explored.

## 4 Discussion and Conclusion: “Do as I Say, Not as I Do?”

This mixed methods study has provided a two‐phase exploration of the stress experiences of stress experts. It aimed to explore the extent to which those with expert knowledge apply this in the management of their own stressful encounters, and how they account for the influence of their expert status. This is an important research gap given the reliance on uptake of skills, awareness, education and knowledge as tools for management of work‐related stress in any workplace population, and we add to the body of work that asks for further consideration of “personal and socially influenced perceptions” (Jennings‐ Black and Britt 2022, 2) of stress and health experiences. Exploring the experiences of experts in work‐related stress also allows a unique perspective in responding to calls for work that further evaluates stress initiatives and how learning from an intervention is applied and reflected upon longitudinally over time (Nielsen and Shepherd 2022). Indeed, stress experts can by their definition be termed exemplary in their acquisition of such knowledge, but how such learning is applied or transferred is not known.

Phase one found that stress experts experienced less stress than a norm group. Objective experience in terms of length of time as an expert did not influence the relationship between stressor exposure and outcomes. Belief in the influence of expert status did impact outcomes. This is especially interesting as it develops our theoretical understanding of how beliefs about stress and awareness can impact more readily than the content of the interventions themselves and extends the work of scholars such Jennings‐ Black and Britt (2022) who have sought to explore damaging or limiting beliefs. Phase two unearthed a number of narrative themes that helped to illuminate the complexities in how expert status is deployed as the stress expert navigates their work and life. These life history narratives provided examples of spirals of learning regarding the intersection of emotions, cognitions and behaviors.

We support and extend the theoretical insights in to how learning about well‐being and health is applied experientially over time. Expert status is seen to continually evolve over time as the expert consolidates their learning further through an examination of their own stress, their ever‐developing theoretical understanding, and the stress experiences of those they work with. These interactions work to perpetuate, develop and change one's beliefs about their purpose as it is woven with their identity. This provides theoretical support for work that examines the intricacies of calling in emotion work and impacts on health (Andel et al. 2022). We argue that a cyclical framework such as the ITTEM model proposed by Nielsen and Shepherd (2022) helps to support the nuanced understanding of how expert status is made sense of. We suggest that our data provides evidence of phases 4 and five of the ITTEM model in action—where we can evidence cognitive and emotional shifts that are referenced by the expert exploring their previous and current experience. Though our data in Phase 2 is collected at one time point, we apply a temporal examination of it given our focus on life histories at the within person level.

Our theoretical framework integrated a number of principles in addressing research gaps and recent research agendas for understanding stress beliefs and how they impact behaviors. Mixed methodology enabled the complexity in lived experience to be accessed at both the nomothetic and idiographic level. We saw examples within the narrative themes where spirals of learning and transfer of knowledge are abandoned or set aside as a function of potent beliefs that have the potential to derail intentions to apply healthy coping. Our data illuminates a number of instances where stress experts' transfer of learning is influenced by their changing beliefs about stress as their knowledge interacts with their environmental exposure to stressors in different contexts over time, and is sometimes complicated by paradox. For example, in the narrative theme of overwork, where stressors are appraised as welcome or enjoyable they are seen to perpetuate further overwork. Where they are seen as damaging in light of different factors (for instance ‐ one participant acknowledges the harm) we see changes to cognition that have the potential to shape more favorable outcomes. We therefore contribute to work such as that of Kirrane et al. (2018) that signposts the value in qualitative enquiry for exploring complexities in overwork. Although these paradoxes are often embedded within stress expert identity expectations, we suggest they have wider resonance for any individuals within busy roles where the balance between working hard and protecting one's health presents a tension. Practicing what one preaches is for some part of an important exercise in self‐image and credibility as well as a health‐protecting behavior and we support the work on callings in exploration of the factors that sit below meaning in such roles and impacts on health (Zhou et al. 2023; Andel et al. 2022).

Our data also provides examples of how such transfers of learning are shaped over time. By examining life histories, motivations for becoming a stress expert we uncover some belief systems that sit below experts' behaviors—for example for some, the need to help others is only credible if one can embody the stress management techniques themselves. Evidence of learning spirals are also found where participants recall how their coping or approach to their own stress is influenced not only by their knowledge of it, but how they have learnt in previous times of difficulty, as in the example of the participant who cites a period of time in which many life changing and traumatic events took place. There were also interesting nuances in how beliefs are static or malleable—we saw examples of sensemaking that were deterministic where some participants held a belief that predisposed elements of their personalities were deeply intertwined with behaviors and therefore perceived as resistant to change despite their knowledge about stress, and examples of stress as pervasive and fatalistic.

Our findings also contribute to the small body of literature on expert status. We add to the emerging work that examines health professionals' interaction of their own identities alongside their role of helping others (Kranabetter and Niessen 2017; Preston 2022; While 2015). Of particular note is the emergence of this dynamic as a possible stressor—indeed, the need to manage one's own stress to perpetuate a credible identity is challenging, especially alongside experiences of stress as ubiquitous, heightened workloads, and for some a preference for engaging in overwork (Kirrane et al. 2018). Our data therefore begins to disentangle the complexities, conflicts and paradoxes in ‘practicing what one preaches’—indeed, this is not a simple decision for the stress expert. We see both healthy and unhealthy coping strategies employed together, tensions about being authentic in sharing one's own experiences against exposing vulnerabilities and impacting on credibility, and beliefs that sometimes stress and its outcomes are unavoidable. Taken together, it is apparent that social expectations of others in response to expert status is an important contextual factor that impacts stress identity expectations (Burgman et al. 2011). We argue this presents a number of challenges in managing expert status.

In returning to an understanding of stress prevalence in the expert population, we suggest that perhaps stress experts report less stress and fewer impacts on their psychological health than the norm group in study one due to their unique appraisal of stress as a function of their expert status. As Casper et al. (2017) suggest: “a situation is appraised as a challenge if it is associated with a potential for mastery, growth, or gain” (p799). Stress for stress experts is possibly framed differently in terms of its potential as a learning experience. We know that exposure to stress for some of our participants was seen as further learning (Sawhney et al. 2018) and a valuable contribution to their ‘toolkit’ that can act as a further spiral for how application of such knowledge helps the stress experts themselves in the future, and indeed those who they support in their work. The very presence of stress provides ongoing learning opportunities for the stress expert, is by some seen to impact their credibility (in both positive and negative ways), and this was referenced by participants across the different thematic areas of our analyses. It could also be expected that stress experts may rate stress impacts as less due to the aforementioned identity expectations, though stress experts were happy to disclose examples of highly stressful situations and outcomes during the interviews.

Taken together, our contribution provides theoretical insights in to how stress knowledge is utilized in situ, and how appraisal of it moves far beyond the momentary context the knowledge is acquired within, and instead evolves continuously as a function of context and environment, motivations, and spirals of learning. This, we assert, illuminates the theoretical and practical relevance of the process orientation of stress experiences within their entirety and as fluid and ever‐shifting encounters.

### 4.1 Implications for Practice

Our data suggest that beliefs concerning the influence of one's expert status regarding stress knowledge, as opposed to the objective measurement of one's skills and experiences, is more potent in shaping outcomes. We argue that this will have important implications for practice in the enactment of employee stress and health interventions. Indeed, in terms of optimizing efficiency in employee level interventions such as stress management training, practitioners may want to ensure that the content allows for detailed exploration of employee beliefs and preconceptions about stressful circumstances, including expectations and beliefs in the influence of these upon on their workplace experiences and well‐being. This could, for example, take the form of introspection through critical reflective practice activities where time and space is provided for employees to reflect upon not only their experiences of stress but their underpinning beliefs and values about it. Further research could develop toolkits or resources based on our model to explore key themes arising from our data. We suggest these are relevant not only in stress training in a broad sense, but also in terms of education and awareness regarding influences on stress coping. Beliefs about stress and one's knowledge of it could be measured or explored through the use of scales that could feature in stress audit measures, and could have wider resonance in unpicking positive or damaging organisational cultures and climates, also building on the work of others (Jennings‐ Black and Britt 2022). This may help in ensuring return on investment through heightened training effectiveness, and may also work to support the development of positive organisational cultures and climates through for example, role modeling and shared learning.

Role modeling and its role in the development of positive occupational health climate and culture warrants further investigation, where the socialization of expectations and norms around stress within an organisation could be explored in group activities or guided workshops. Examples from our data illuminate how experts were mindful of their need to model healthy behaviors for their peers despite often themselves working in unhealthy ways. This is relevant to any environment where health outcomes are pertinent besides that of stress experts. For example, how might the credibility of physical health advice be jeopardized by a doctor who themselves appears to engage in unhealthy behaviors? An exploration of the prevalence of healthy and unhealthy role models with regards to wellbeing and impact on employees or service users in health settings would be a fascinating avenue for further research alongside the development of content for reflective practice within stress and well‐being training. This could consider, for example, the values and behaviors of *both* leaders and employees, the consistency or inconsistency across these (perhaps by way of a gap analysis), and how they influence perceptions of stress experiences and perpetuate or prevent behaviors such as overwork. Drawing on culture or climate measures as well as stress audits and integrating the two together in organisational initiatives would be beneficial. Similarly, the consideration of role modeling behaviors could be included alongside or in addition to more traditional stress audits and the development of new tools to measure this would be a fruitful avenue for further research and practice.

In sum, we therefore suggest that a focus within intervention training with regards to beliefs is needed to ensure the development of productive stress mindsets and positive outcomes.

Our findings are also relevant for stress experts themselves (academics, practitioners or organisational leads for stress, health or well‐being initiatives) as gate keepers, and we suggest that those responsible for stress management can benefit from understanding the challenges in managing one's own stress experience and identity within a role that positions them as an advocate for stress management and responsible for the perpetuation of occupational health culture and climate—especially in terms of the complexities that impact leading by example (While 2015). Though here we explore the experiences of academic stress experts, we suggest our findings may be useful in contributing to debates about how stress or wellbeing experts within organisations navigate their own stress experience, disclose their own coping and vulnerabilities and build a culture of shared learning and understanding.

### 4.2 Limitations and Future Research

Our study made use of a self‐report survey of participants' own stress in reference to expectations about stress expert identity. We acknowledge the limitations of the cross‐sectional nature of study one and the challenges for causality as a function of both the possibility for reverse causality between attitudes and well‐being, and also potential social desirability of participant responses given their status as experts. We acknowledge that multi‐source perspectives—for instance, considering others' perceptions of stress experts' identity may be useful, and that response biases in the self‐selection to the research study is likely (Krause 1985). We also acknowledge alternative statistically modeling approaches that may be used to explore the potential bi‐directional nature of the relationships between variables. We further suggest that longitudinal data would be valuable in exploring the trajectory of experiences and respond to calls for an examination of time (Casper et al. 2017; Spencer et al. 2021). Because we asked experts to recount historical motivations for developing their career in stress, and their responses provided situational and behavioral examples that took place at different time points as they recounted such experience, we were able to illustrate how transfers of learning took place and appraise the process orientation of stress, but we acknowledge this relied on retrospective narrative accounts (Crozier and Cassell 2016).

There are other potential theoretical frameworks that could be used as a lens to further extend the interplay between identity elements and stress experiences for the stress expert. For example, it appears that many of the beliefs examined could constitute threats to identity for the stress expert, and an examination of further identity constructs such as identity work and regulation could be helpful, especially in terms of developing the theoretical underpinning of expert status and identity as an expert.

## 5 Conclusion

Stress awareness and education although very important in informing the analysis of one's stressful circumstances, appear on their own to lack enough power to shape outcomes. Indeed, for stress experts this was explored in terms of beliefs about the prevalence of stress, and how the mechanisms underpinning awareness are made sense of, reflected upon, and translated in to changes in behavior. This has far‐reaching impacts for stress management interventions where more knowledge is needed concerning the factors that shape success of such interventions (Nielsen and Shepherd 2022). We argue that education about stress and health is not enough to equip individuals to cope with exposure to stressful work situations—even for stress experts. We echo the calls of other researchers to develop further our understanding of beliefs about stressful experiences (Casper et al. 2017; Jennings‐ Black and Britt 2022; Kirrane et al. 2018). Our findings illuminate some of the process‐orientated factors that sit within stress experts' experiences as a function of their sense of purpose in their role and wider identity (Andel et al. 2022). Taken together, these present complicated challenges for how learning is reflected upon and applied in one's own management of stress as a function of beliefs, previous and current experience, and ongoing shifts in learning from context and environment.

## Conflicts of Interest

The authors declare no conflicts of interest.

## Supporting information

Supporting Information S1

## Data Availability

The data that support the findings of this study are available on request from the corresponding author. The data are not publicly available due to privacy or ethical restrictions.
