# Supplementary material for: Do We Practice What We Preach? A Mixed Methods Study of Stress in Stress Experts: Implications for Transfer of Awareness and Learning
Source: Stress Health. 2025 Jun 20;41(3):e70064. doi: 10.1002/smi.70064 (PMC12180487; doi:10.1002/smi.70064)
Supplement: Supplementary file 1 — Supporting Information S1 [file SMI-41-e70064-s001.docx]

**Supplementary files – qualitative analysis**

| **Analytical segments** | **Emergent high level nomothetic narrative themes** | **Quotes** |
| --- | --- | --- |
| Motivations for becoming a stress expert | Personal exposure as informing career choice | *“I do suppose it has been a bit of cycle really, because if it wasn’t for the extreme stress and trauma then I wouldn’t have chosen the career… but my understanding of the brain has allowed me I suppose to forgive myself for not having been able to manage stress…*  *“I think that people who work in the stress field are people who do have stress issues…it is not coincidental what you choose to do…I don’t believe that anybody picks a job that doesn’t have some psychological underpinning to it”.*  *“But when you unwrapped it you realised it was the stress, the expectation and pressure that would be put on them…the stress of moving their families…it was several layers of stress, and so we unearthed that, but when I got there I started to think ‘hmm some of the interviews that I am hearing are me’ so you know, so that’s when the personal came in to it”.* |
|  | Helping others | *“I used to gravitate towards other people’s crisis and stuff because I wanted to know that I had a role and a function, and I knew that I could take it and I could absorb the stress, well I thought that I could. I thought that that was my role, I had inherited it, this profession, my role on the planet was to put people’s stress, to absorb other people’s dysfunction, and it was only through addressing why I had kind of learnt that kind of thing when I was younger”.* |
| Experiences and awareness of stress | Stress experts are not immune to stressful experiences and damaging consequences | *“Having had a couple of anxiety periods when I have had to have medication and it surprised me and it sort of shocked me, I am really now very, very careful about my sleep. If I feel myself not sleeping well, I will take a day off work, if I have had three or four nights where I haven’t slept, I go ‘okay I am going to stay home and actually rest’… that is one of the things that I have put in place really as a thing that I do now, I just really watch my sleep, because sleep I think is, is so critical managing stress”.*  *“When I had those two acute episodes of stress, I was alarmed that I could actually get to that point and that I had such a lack of self-care, of course I reflect upon the fact that three years ago when [several stressful life events took place]. I don’t know if I could have done very much about it, you know, it just happens. It’s just a situational acute stressor, but I guess day to day I can manage my stress I think much better now than I could three years ago”.* |
|  | The stress expert as overworking (I do not practice what I preach? | *“And it is my fault sometimes…I know how hard it is and I hate this trite stuff when you say to people ‘oh you know, just stop work at five o’clock and have a life and this that and the other’ because you know how hard it is when you are deeply involved in what you are doing and passionate about it and so I sometimes feel a bit of a fraud really. But it is a difference between the psychological flow and the being in a trapped situation, if you are in the zone loving work, why wouldn’t you carry it on?”.*  *“…if I want to read my emails and send some emails on a Sunday at eight o’clock, so that I can get them out of my head, I will… you know again, with growing knowledge of impact on other people, role models whatever, you have got to think about the impact on the recipient as well…,so I am much more aware of that, and the signals that you are giving to other people, because basically you know, you are communicating implicitly I suppose expectations … [that] you know, the only way to be successful in academia essentially is not to have a life. And you have to be really careful about that”.*  *“…because I do a lot of self-awareness work with others [I know] I can’t disentangle my knowledge about personality… so work to me, there are no boundaries between work and life, because if I want to take work on holiday, why shouldn’t I? I might be bored of the holiday, take a day off in the middle of the week, why shouldn’t I?”*  *“I think that is how I approach most things, I am not ever going to stop, if I am stressed I have got every right to feel stressed sometimes, but I need to understand what triggers it, what the key is what will stop me. I can’t stop myself because sometimes my character makes me take on too much. I always take on too much and then I reach a point and then I have to have a holiday, that is how it is”.*  *“And it is important not to feel like a victim as well, because you know I do a lot of work on work-life balance, but I can tell you that my work life balance is pretty crap... because I kind of choose that”.*  *“I do get stressed, I use work as a coping mechanism and if anything happens to me, you know like really bad things, like my mum died, and it was a very traumatic situation …if things become tough I throw myself into work and I become pretty much incommunicado and I like to insulate myself from the outside world, you know, which I know is not good… so I am pretty much self-managed because I don’t really have a line manager, you know, I just get on with it myself, which means that I work long hours, I am obsessed by work and I use it as coping, and I know that is not healthy at all”.*  *“I take on far too much, I can’t seem to learn not to, even though I know what is going to happen. I can see it all unfolding and actually, this is quite an appropriate time at the moment, because I looked at my diary last night and I kind of got into this feeling of panic… and it is absolutely crazy, but knowing that I am putting myself under pressure, doesn’t stop me from doing it, because there are so many interesting things out there and even though you know that you are going to be busy, and the little voice in your head, you know, what you would tell other people or your clients, you know, ‘look in your diary, you know, pace yourself, you know put in lots of space, you know, thinking space whatever’, I can’t seem to do it myself, and then of course you get to the stage where you get over tired and over committed, erm and that is a bit of a problem”.* |
|  | Reflecting on coping complexities | *“I think that my first approach I suppose when I am confronted by something and it doesn’t always happen immediately but is to try and reflect on what is the real problem here, and what is the cause of the problem and ask myself, can I do anything about this? If the answer to that is yes, then I have to think do I really feel like doing anything about it, do I think that I have the resources to deal with it and if so try and do something obviously whether that is going to work or not is another part of the question, on the other hand if it is not a very serious problem, not a serious sort of stress, or it is one that you feel as though you don’t have any control of it all then I think some kind of avoidance or trying to put it out of your mind is probably more effective than dealing with those kind of things. You don’t want to be worrying if it is not going to make a huge kind of difference to you, your life”.*  *“I can usually pull back, I will take some time, use breathing but also, just try and create some space around the things that are stressing me, see them for what they are, create some mental space, get a bit of perspective, try and imagine them in comparison, in the great scheme of things…. [I] try to understand that it is natural that I would get stressed in these circumstances and that everyone gets stressed and it is not going to kill me”.*  *“I mean working with loads of people over the years, my belief is that none of us are actually worried about anything, or stressed about anything, we are stressed by our emotional reaction to it. I mean, if you thought, I could cope with a marriage break up it is self-efficacy, then I will be able to [cope], but if you think ‘oh my god, I will freak out and I will never be able to face anybody’… I think we are more worried about our emotional response to things rather than the actual thing itself? You know I tend to look at anything, I suppose, when things happen that are stressful, I suppose I do a lot of reflecting on it and what I learnt from it, what can I gain from it, how has it added to my tool kit”.*  *“… the biggest tell really is that’s happening is my relationship with stress, so I am not saying that I don’t get stressed anymore, but I just allowed my brain to develop the capacity to find a tiny window to choose how I respond to that stress signal, and sometimes I don’t get a chance and I can’t do much about it, the stressor was too grand or it is connected to other things and I will feel stressed, but I am aware that I have responded in that way. And just that awareness takes a little bite out of it I think and then there are various things that I do, I suppose, when I notice that I am stressed, either because I have noticed that my thoughts, my patterns are becoming very erratic and speeded up. It is usually around things that I have to do, so it is forward planning and, or if there is someone that is emotionally reliant on me and I am helping and that is really in a crisis, and I need to focus on them plus I have got these other pressures coming in from work and stuff, that is a time when I am likely to feel those stressors”.*  *“We are aware of the transient nature of coping;, one morning you will wake up and you will think - my gosh, there are all of these things that I have to do and it is like ‘tick, tick, tick’, and everything gets filed away beautifully, you know you are really productive and you do really well, l and of course on that day you would rate yourself as being really good at coping with stress, really productive. And then the next day you wake up feeling, you know, a bit low and feeling as if the whole weight of the world is on you and feel pretty crap - I should be able to cope with this, but really that is tricky isn’t it? As we know with stress research that a lot of the measures that we use tend not to capture that because they are done by asking people for a general picture, you know - how do you behave? How do you feel normally? So, you know, sometimes it makes me question the validity of the measures that we are using for that reason, we are forcing people to aggregate”.* |
| Beliefs about expert status | Stress knowledge and education as helpful | *“I suppose if you have the ability to recognise your understanding of stress, then I guess you have the ability to, to know to do something about it. So, it is logical that you then take that step to do something about it. If you were from a position of ignorance, and you didn’t know why stress occurred and where it comes from, and you were just feeling stressed then without that understanding then you don’t feel like that you have got the tools to do anything with it”.*  *“…the more that I use my understanding in my so called expertise about the brain and trauma to demonstrate that it can have a positive effect the better, so I suppose managing my stress in a way isn’t just for me - it is a way for demonstrating that things can be better for other people too, so the more that I do it the more that I am likely to be able to help other people and also for the people that live and work around me, I want to be a person that that is nice to be around”.* |
|  | Heightened awareness and expertise don’t necessarily improve outcomes | *“So having studied up on all these things and done research on them I guess I was able to think about it in a more coherent kind of way… I would have to say that I am not sure that any of this did me a whole lot of good though, in terms of when there were times when I felt very stressed out in the work that I was doing. And even though I was aware of it, there were some times when I just felt that I wasn’t able to change what was happening so that in itself was one of the stressors”.*  *“I think nowadays I feel that I am a lot more attuned to it and aware of it than I was maybe 30 odd years ago. But there are times when… it can kind of creep up on you and you suddenly realise that you are all stressed out as a result of things that have been happening to you or events in your life that you haven’t been able to control. So I think it can go either way, but certainly I guess I would like to think that having researched within this area for [many] years, that I am probably a bit more aware than maybe other people”.*  *“I think awareness and knowledge about different coping strategies and an awareness of things like control, which we talked about before and how important that can be, I think yes, I think an awareness of those things does help, but I think a lot of it does come back to how much stress are you under and what kind role you are performing, and if you are in a very stressful role then knowing about these things might not actually be a huge benefit to you. It will help some, but you are still going to be stressed out”.*  *“I also do a lot of self-awareness goal setting stuff that I have moved into, I also understand my own personality and what triggers those responses in me, and I know what they are but I also can’t always stop them happening...”.* |
|  | Identity and the disclosure paradox for the stress expert | *“But having high expectations of yourself is problematic because you feel that you shouldn’t be feeling like this and you should be able to cope with it in some ways, you know”.*  *“I used to feel that I am a bit of a fraud because I was young, I didn’t have enough experience of stress to be able to understand it, and then of course through your life when you do experience things that are stressful, I started to think that at least I felt more authentic…”*  *“…once I gave a workshop…anyway, this person introduced me and said – [name] is going to talk about stress, and having had the pleasure of her at lunch I realise that she is the least stressed person I have ever met’. And I remember thinking ‘is this an insult?’ and actually it was a compliment saying, you know, she obviously practices what she preaches…you can theorise about it but should also look like somebody practicing what you preach. Why would you talk about this stuff if you know, you can’t do it [yourself]? But the flip side is that I was doing a session last week and I used some bits of personal disclosure and in that I thought ‘I hope they don’t think that I am actually a very vulnerable or weak person’ … so there is this balance all the time and it can be quite tricky”.* |
|  | Learning from others | *“I mean working with loads of people over the years, my belief is that none of us are actually worried about anything, or stressed about anything, we are stressed by our emotional reaction to it. I mean, if you thought, I could cope with a marriage break up it is self-efficacy, then I will be able to [cope], but if you think ‘oh my god, I will freak out and I will never be able to face anybody’… I think we are more worried about our emotional response to things rather than the actual thing itself? You know I tend to look at anything, I suppose, when things happen that are stressful, I suppose I do a lot of reflecting on it and what I learnt from it, what can I gain from it, how has it added to my tool kit”.*  *“I find myself switching from a personal to a professional persona almost instantaneously sometimes and also being involved in health psychology that happens when you engage with health care, because you are watching the doctor-patient communication and you are thinking ‘ooh, that’s really good, that’s really talented, he’s doing that really well’…and just flipping back and forth between expert and individual I think that is quite an interesting place to be because of course I think the personal and professional is actually melded very much, yet obviously you don’t spill your heart out to students or whatever, but you can draw on these experiences”.*  *“I don’t believe I am better at coping because of the knowledge I have of it, I think I am better at coping from what I am hearing from ordinary people. That is where I am learning, I am not learning from the science, the science is telling me to have more control, to have better balance, no - I have heard stories of how balance damages people and how long hours damages, how imbalance damages people, that is what I am learning…it is lived experience of listening to people and that is what psychologists should do I think” .* |
